# The effect of varying levels of purified condensed tannins on performance, blood profile, meat quality and methane emission in male Bapedi sheep fed grass hay and pellet*-*based diet

**DOI:** 10.1007/s11250-022-03268-7

**Published:** 2022-08-12

**Authors:** J. W. Ngámbi, M. J. Selapa, D. Brown, T. G. Manyelo

**Affiliations:** Department of Agricultural Economics and Animal Production, University of Limpopo, Private Bag X1106, Sovenga, Polokwane, 0727 South Africa

**Keywords:** Bapedi sheep, Condensed tannins, Sheep performance, Meat quality, Methane emission

## Abstract

This study determined the effect of purified condensed tannin inclusion levels in a diet on production, haematological indices, blood biochemical components, meat quality and methane emission by yearling indigenous male Bapedi sheep on a grass hay and sheep pellet-based diet in a 28-day trial. The diets contained similar (*P* > 0.05) nutrients but with different (*P* < 0.05) purified condensed tannin supplementation levels. A complete randomized design was used. Twenty-four yearling male Bapedi sheep were assigned to four dietary treatments having different purified condensed tannin levels of 0 (GH_80_P_20_PCT_0_), 30 (GH_80_P_20_PCT_30_), 40 (GH_80_P_20_PCT_40_) and 50 (GH_80_P_20_PCT_50_) g/kg DM. A quadratic type of equation was also used to determine condensed tannin supplementation levels for optimal performance and methane emission reduction by sheep. Supplementing diets with purified condensed tannins did not affect (*P* > 0.05) diet intake, digestibility and live weight gain of male Bapedi sheep. Supplementing diets with purified condensed tannins did not affect (*P* > 0.05) blood components of male Bapedi sheep. Inclusion of condensed tannins in the diets did not affect (*P* > 0.05) Bapedi sheep meat pH and sensory attributes. However, supplementing diets with purified condensed tannins decreased (*P* < 0.05) methane emission by 51 to 60%. A 49.08 g supplementation level with purified condensed tannins per kg DM diet was calculated, with the use of quadratic equations, to result in the lowest methane emission by male Bapedi sheep. The meat of male Bapedi rams on diets containing 30, 40 or 50 g of purified condensed tannins per kg DM contained higher (*P* < 0.05) antioxidant activities than those from rams fed a diet without purified condensed tannins. These results indicate that purified condensed tannin supplementation levels of 0, 30, 40 or 50 g/kg DM diet had no adverse effects on growth performance, blood profiles and meat sensory attributes of male Bapedi sheep. However, supplementation levels of 30, 40 or 50 g of purified condensed tannins per kg DM diet reduced methane emission by 51 to 60%, and increased sheep meat antioxidant activity values. Supplementing diets with purified condensed tannins has the potential to reduce methane production and emission by sheep. However, long-term studies are recommended to ascertain the present findings.

## Introduction

The Bapedi sheep came with the Bapedi people who migrated southwards into Limpopo Province and settled in Sekhukhune, Limpopo Province of South Africa between 200 and 400 AD (Hlophe, 2011). It is important for milk, meat, hide and skin (Snyman, 2014). However, methane (CH_4_) produced by ruminants adversely contribute to climate change (Lassey, 2007; Kaufmann et al., 2006). About 80 million tons of methane are produced annually, where 47% of those emissions are contributed by agriculture and 39% of that is emitted by ruminants through the digestive processes (Lassey, 2008; Eckard et al., 2010; Gerber et al., 2013). Eighty-five percent of the methane produced by ruminants is from enteric fermentation, with cattle, buffalos and small ruminants contributing 77, 13 and 10%, respectively (Gerber et al., 2013). Methane emission results in a 12% loss of energy intake by ruminants (Johnson and Johnson, 1995). Tannins in a diet inhibit ruminal ammonia and methane production during microbial fermentation (Carulla et al., 2005). Low tannin intake levels reduce methane production and emission without any adverse effects (Makkar et al., 2007). Various studies indicate that dietary tannins improve meat quality (Ngambu et al., 2012, 2013; Mapiye et al., 2009). However, Mapiye et al. (2010) reported no improvement in the meat quality of the animals on tanniniferous diets. Tannins are anti-nutritional factors and, hence, when taken in large amounts they can be toxic to the animal (Olafadehan et al., 2014). Such effects are manifested in haematological and biochemical indices (Taiwo and Anosa, 1995). However, data on purified condensed tannin supplementation levels for optimal methane emission reduction, meat quality and blood profiles of Bapedi sheep is not extensive and conclusive. This study, therefore, focused on the effect of supplementation level of purified condensed tannins in a diet on production, haematological indices, blood biochemical components, meat quality and methane emission by yearling male Bapedi sheep fed a diet based on grass hay and sheep pellets. Such information is useful in formatting strategies for improving sheep production and reducing their methane emission.

## Materials and methods

### Study site, experimental design, diets and data collection

The experiment was done at the University of Limpopo (latitude 27.55°S and longitude 24.77°E). The area is semi-arid, and the vegetation is mainly grass species with mixtures of shrubs and trees. The shrubs are mainly tanniniferous Acacia species (Shiringani, 2007). Twenty-four yearling male Bapedi sheep, weighing 25 ± 1.6 kg, were allocated in a completely randomized design to four treatments having different purified condensed tannin supplementation levels of 0 (GH_80_P_20_PCT_0_), 30 (GH_80_P_20_PCT_30_), 40 (GH_80_P_20_PCT_40_) and 50 (GH_80_P_20_PCT_50_) g/kg DM (Table 1). The experimental diets were isocaloric and isonitrogenous. The experiment lasted for 28 days (21-day preliminary and 7-day collection periods). The sheep were accommodated in well-ventilated individual metabolic pens (1.4 m × 2.0 m) with separate compartments for faecal and urine collections. Feed was offered ad libitum, allowing a 15% refusal (Kaitho et al., 1996). The purified condensed tannins were purchased from Bondite Limited, South Africa. The animals were offered ad libitum water and a mineral lick (Table 2). An ethical clearance certificate (Certificate No: AREC/07/2019: PG) was obtained before the start of the experiment.

**Table 1:** Dietary treatments for the study

Experimental code	Treatment description
GH_80_P_20_PCT_0_	A mixture of 80% grass hay and 20% pellets without any purified condensed tannins
GH_80_P_20_PCT_30_	A mixture of 80% grass hay and 20% pellets plus 30 g of purified condensed tannins/kg DM feed
GH_80_P_20_PCT_40_	A mixture of 80% grass hay and 20% pellets plus 40 g of purified condensed tannins/kg DM feed
GH_80_P_20_PCT_50_	A mixture of 80% grass hay and 20% pellets plus 50 g of purified condensed tannins/kg DM feed

**Table 2:** Nutrient composition of the mineral block

Nutrients	Quantity	Units
Protein (min)	200	g/kg
Urea (max)	22	g/kg
Crude fibre (max)	100	g/kg
Calcium (min/max)	10/35	g/kg
Phosphorus (min)	4	g/kg
Moisture (max)	140	g/kg
Sulphur	8	g/kg
Potassium	12	g/kg
Copper	15	mg/kg
Manganese	100	mg/kg
Zinc	120	mg/kg
Cobalt	0.5	mg/kg
Iodine	3	mg/kg
Iron	75	mg/kg
Selenium	0.5	mg/kg
Vitamin A Energy	88 8.8	IU/kg MJ ME/kg

Source: Voermol Maxi Block, South Africa

Daily diet intake and weekly live weights were determined during the collection period (McDonald et al., 2011). All faeces were collected and apparent nutrient digestibility was determined (McDonald et al., 2011) during the collection period. Blood was collected from the jugular vein of each animal on days 21 and 28 of the trial into sample bottles. A hand-held methane detector was used to measure methane emissions (Chagunda et al., 2009). Measurements were taken at 8.00 h in the morning. Methane emitted was reported as parts per million-meter (ppm-m) (Chagunda et al., 2009).

All the sheep were slaughtered at the end of the experiment and their meat was subjected to sensory evaluation (tenderness, flavour and juiciness), pH and colour to determine meat characteristics. The meat was also subjected to colour and shear force determination (Instron Universal Testing Machine, Model 3344, Instron Industrial Products, GC, USA). The method adopted by Pavelková et al. (2013) was used for the sensory evaluation of sheep meat. The following sensory attributes were evaluated by the sensory panel: tenderness, juiciness and flavour of meat samples. The sensory panel consisted of 20 trained panellists. Each panellist was offered to drink lemon juice after tasting meat from each treatment before proceeding to the next treatment to wash out the previous treatment to avoid confusion of tastes. The five-point ranking scale scores used are indicated in Table 3. Nothing was added to the meat samples so as not to affect the taste. An oven set at 105ºC was allowed to preheat before cooking. The meat samples were put in trays and they were covered with aluminium foil to prevent water loss. Thereafter, the trays with meat were put in an oven for approximately 60 min and the meat samples were turned after every 10 min. Samples were cut into small 5 cm cubic pieces and served immediately after cooking.

**Table 3:** Evaluation scores used by the sensory panel

Sensory attribute			
Score	Tenderness	Juiciness	Flavour
1	Too tough	Too dry	Very bad flavour
2	Tough	Dry	Poor flavour
3	Neither tough nor tender	Neither dry nor juicy	Neither bad nor good flavour
4	Tender	Juicy	Good flavour
5	Too tender	Too juicy	Very good flavour

Source: Pavelková et al. (2013)

### Chemical analysis

AOAC (2012) procedures were used to determine the nutrient composition of the diets. The methods of Van Soest (1994) were used to determine neutral detergent fibre (NDF) and acid detergent fibre (ADF). A bomb calorimeter was used to determine the gross energy of diets and faeces (AOAC, 2012). Butanol-HCl method was used to determine condensed tannins (expressed as leucocyanidin equivalent, % DM) (Makkar et al., 1995). The blood samples were analysed for complete blood counts and blood serum metabolites. Total white blood cell numbers were determined by the method of Natt and Herrick (1952). The lymphocyte population was evaluated from stained blood smears with brilliant cresyl blue (Mukkur and Bradley, 1974). The procedures outlined by Dacie and Lewis (2001) were used to determine haematocrit and haemoglobin concentrations. Neubauer haemocytometer was used to determine red blood cells and total white blood cells. Serum protein was determined (Valley et al., 1980). Enzymatic colorimetric test (Quimica Clinica Applicada, SA Kit) was used to determine blood glucose. Minerals were estimated by atomic absorption spectrophotometer, Model 490 (Gallenkamp and Co. Ltd., London). The methods of Reitman and Frankel (1957) and Roy (1970) were used to measure the activities of the enzymes. The 2, 2-diphenyl-1-picrylhydrazyl (DPPH) method was used to determine antioxidant activities in meat (Sgherri et al., 2012).

### Statistical analysis

A one-way analysis of variance using the general linear model procedures of SAS version 9.1.3 (SAS, 2008) was used. Statistical model used was as follows: *Y*_ij_ = *µ* + *T*_i_ + *e*_ij_, where *Y*_ij_ = overall observation, *µ* = overall means, *T*_i_ = treatment effect and *e*_ij_ = residual error. The least significant difference test was used to test the significance of differences between means (*P* < 0.05; SAS 2008). The quadratic equation was used to determine purified condensed tannin levels for optimal methane reduction by Bapedi sheep (SAS, 2008):

$$Y = a + {b}_{1}x + {b}_{2}{x}^{2} + e$$

where *y* = overall observation; *a* = intercept; *b*_1_ and *b*_2_ = coefficients of the quadratic equation; *x* = level of purified condensed tannin supplementation; − *b*_1_/2*b*_2_ = purified condensed tannin supplementation level for optimal response; and *e* = error.

## Results

The nutrient contents of the diets were similar (*P* > 0.05) except for purified condensed tannins (*P* < 0.05) (Table 4). Supplementation with purified condensed tannins increased (*P* < 0.05) the condensed tannin contents of the diets.

**Table 4:** Experimental diet nutrient composition

Nutrient (%)	Diet^#*^			
	GH_80_P_20_PCT_0_	GH_80_P_20_PCT_30_	GH_80_P_20_PCT_40_	GH_80_P_20_PCT_50_
Dry matter (%)	95^a^ ± 0.53	94^a^ ± 0.70	95^a^ ± 0.55	95^a^ ± 0.57
Organic matter (%)	86^a^ ± 0.52	86^a^ ± 0.07	86^a^ ± 0.44	86^a^ ± 0.45
Crude protein (%)	12.5^a^ ± 0.65	13^a^ ± 0.58	13^a^ ± 0.53	13^a^ ± 0.51
NDF (%)	45^a^ ± 0.48	45^a^ ± 0.03	45^a^ ± 0.25	45^a^ ± 0.21
ADF (%)	35^a^ ± 0.23	35^a^ ± 0.44	35^a^ ± 0.32	35^a^ ± 0.38
Fat (%)	2.8^a^ ± 0.31	2.8^a^ ± 0.25	2.8^a^ ± 0.13	2.8^a^ ± 0.26
Energy (MJ/kg DM)	18.3^a^ ± 0.26	18.4^a^ ± 0.19	18.2^a^ ± 0.21	18.7^a^ ± 0.32
Condensed tannins^#^	0^d^ ± 0.00	30^c^ ± 0.01	40^b^ ± 0.03	50^a^ ± 0.02

^*^Values presented as Mean ± standard error (SE)

^a,b,c,d^Means in the same row not sharing a common superscript are different (*P* < 0.05)

#Treatments were supplemented with 0 (GH_80_P_20_PCT_0_) 30 (GH_80_P_20_PCT_30_), 40(GH_80_P_20_PCT_40_) or 50 (GH_80_P_20_PCT_50_) g of purified condensed tannins per kg DM diet. Diet^#*^: 0, 30, 40 or 50 g of condensed tannins per kg DM diet

Supplementing diets with purified condensed tannins did not affect (*P* > 0.05) feed intake, digestibility and growth of yearling male Bapedi sheep (Table 5). However, supplementing diets with purified condensed tannins decreased (*P* < 0.05) methane emission by male Bapedi sheep. A 49.08 g supplementation level with purified condensed tannins per kg DM diet was calculated, with the use of quadratic equations, to result in the lowest methane emission by male Bapedi sheep (*Y* = 23.882 +  − 0.58883978*x* + 0.006066*x*^2^; *r*^2^ = 0.999). The supplementation levels of 0, 30, 40 or 50 g of purified condensed tannins per kg DM diet used in this study did not affect (*P* > 0.05) haematological indices, blood metabolites and electrolytes of yearling male Bapedi sheep (Table 6). Supplementation with 0, 30, 40 or 50 g of purified condensed tannins per kg DM had no effect (*P* > 0.05) on male Bapedi sheep meat pH, colour, tenderness, juiciness, flavour, shear force and overall acceptability (Table 7).

**Table 5:** Effect of supplementing diets with purified condensed tannins on intake, digestibility (%), methane emission and performance of male Bapedi rams

	Diet^#*^			
Variable	GH_80_P_20_PCT_0_	GH_80_P_20_PCT_30_	GH_80_P_20_PCT_40_	GH_80_P_20_PCT_50_
DM intake (g/sheep/day)				
DM	265 ± 63.89	338 ± 40.30	368 ± 40.48	287 ± 40.95
OM	230 ± 52.77	297 ± 34.95	320 ± 41.70	250 ± 34.01
CP	18.9 ± 8.21	25.5 ± 3.30	30.1 ± 4.29	25.0 ± 3.60
NDF	161 ± 29.15	195 ± 22.97	214 ± 26.51	166 ± 22.40
ADF	109 ± 30.95	128 ± 19.96	151 ± 23.67	110 ± 25.85
Fat	3.5 ± 0.90	4.5 ± 0.58	5.1 ± 0.77	4.4 ± 0.55
Energy intake (MJ/day)	5.6 ± 0.71	6.0 ± 0.68	6.5 ± 0.72	5.1 ± 0.69
Intake (g/kgW^0.75^)				
DM	35 ± 17.52	53 ± 3.81	55 ± 8.67	46 ± 10.78
OM	33 ± 14.48	49 ± 7.53	50 ± 8.47	43 ± 10.04
CP	4.7 ± 2.07	7 ± 0.24	7 ± 1.36	6 ± 1.47
NDF	16 ± 7.24	26 ± 2.89	26 ± 3.29	21 ± 5.01
ADF	11 ± 4.17	17 ± 2.58	17 ± 2.83	14 ± 3.36
Fat	1.1 ± 0.811	1.1 ± 0.741	2.2 ± 0.571	1.2 ± 0.712
DM	62 ± 0.60	63 ± 1.57	62 ± 0.63	63 ± 1.20
Energy intake (MJ/kg W^0.75^)	0.91 ± 0.091	1.1 ± 0.064	1.1 ± 0.085	1.1 ± 0.174
Digestibility (decimal)				
DM	0.6 ± 0.15	0.6 ± 0.07	0.6 ± 0.08	0.4 ± 0.28
OM	0.6 ± 0.16	0.6 ± 0.07	0.6 ± 0.09	0.4 ± 0.27
CP	0.4 ± 0.35	0.4 ± 0.12	0.4 ± 0.13	0.2 ± 0.48
NDF	0.5 ± 0.20	0.4 ± 0.09	0.6 ± 0.11	0.2 ± 0.38
ADF	0.3 ± 0.29	0.3 ± 0.12	0.4 ± 0.15	0.1 ± 0.53
Fat	0.7 ± 0.52	0.4 ± 0.20	0.4 ± 0.29	0.4 ± 0.92
Energy	0.5 ± 0.18	0.5 ± 0.08	0.6 ± 0.09	0.4 ± 0.34
Methane emission (ppm-m)	23.9^a^ ± 2.80	11.5^b^ ± 1.02	10.3^b^ ± 1.04	9.5^b^ ± 1.02
Live weight (Kg/sheep)				
Initial weight	25.0 ± 0.67	25.1 ± 0.58	24.9 ± 2.03	24.8 ± 2.67
Final weight	25.7 ± 2.31	25.8 ± 0.33	25.6 ± 4.36	25.5 ± 6.00
Wt. gain(g/sheep/day)	25 ± 0.33	25 ± 0.88	25 ± 0.33	25 ± 0.60
FCR	11 ± 2.33	13 ± 1.8	14 ± 2.8	12 ± 1.33

^*^Mean ± standard error (SE)

^a,b^Means in the same row not sharing a common superscript are different (*P* < 0.05)

^#^Treatments were supplemented with 0 (GH_80_P_20_PCT_0_) 30 (GH_80_P_20_PCT_30_), 40 (GH_80_P_20_PCT_40_) or 50 (GH_80_P_20_PCT_50_) g of purified condensed tannins per kg DM diet

**Table 6:** Effects of supplementing diets with purified condensed tannins on blood components of Bapedi rams

Variable	Diet^#*^			
	GH_80_P_20_PCT_0_	GH_80_P_20_PCT_30_	GH_80_P_20_PCT_40_	GH_80_P_20_PCT_50_
Haematology				
Red blood cell (× 10^12^/L)	1.7 ± 4.91	2.4 ± 0.86	2.0 ± 0.78	2.0 ± 0.33
Haemoglobin (g/dL)	9.4 ± 0.92	9.9 ± 0.93	9.7 ± 0.95	8.4 ± 0.92
Haematocrit (L/L)	0.1 ± 0.03	0.1 ± 0.03	0.1 ± 0.03	0.1 ± 0.03
White blood cell (× 10^9^/L)	18.5 ± 10.6	19.8 ± 6.54	29.6 ± 7.72	17.5 ± 6.89
Metabolites (mmol/L)				
Urea	3.3 ± 0.62	3.4 ± 0.58	4.03 ± 0.69	2.5 ± 0.92
Glucose	1.7 ± 1.02	2.8 ± 0.30	2.89 ± 0.21	3.0 ± 0.19
Cholesterol	3.3 ± 0.84	2.8 ± 0.18	2.42 ± 0.13	2.5 ± 0.12
Proteins (g/L)				
Total protein	75.0 ± 2.82	71.3 ± 2.54	68.7 ± 2.50	70.0 ± 2.50
Albumin	24.5 ± 1.97	26.0 ± 1.97	25.0 ± 2.01	28.0 ± 2.00
Enzymes (IU/L)				
Alkaline phosphate	63.0 ± 30.98	85.0 ± 31.20	108 ± 29.52	84.0 ± 30.45
Alanine transaminase	16.5 ± 2.39	18.7 ± 1.83	17.0 ± 1.75	19.7 ± 1.94
Aspartate transaminase	65.0 ± 7.46	69.0 ± 5.26	69.3 ± 6.30	73.3 ± 5.83
Electrolytes (mmol/L)				
Sodium	143 ± 1.32	145 ± 1.42	143 ± 1.56	146 ± 1.33
Potassium	7.6 ± 0.61	6.7 ± 1.61	7.6 ± 0.49	7.1 ± 0.49
Chloride	110 ± 1.72	111 ± 1.52	114 ± 2.41	112 ± 1.32
Total calcium	2.2 ± 0.06	2.2 ± 0.06	2.2 ± 0.06	2.2 ± 0.06
Inorganic phosphate	1.8 ± 0.15	1.8 ± 0.14	2.0 ± 0.15	1.9 ± 0.16
Magnesium	1.0 ± 0.06	1.0 ± 0.06	1.0 ± 0.06	1.1 ± 0.07

^*^Mean ± standard error (SE)

^#^Treatments were supplemented with 0 (GH_80_P_20_PCT_0_) 30 (GH_80_P_20_PCT_30_), 40 (GH_80_P_20_PCT_40_) or 50 (GH_80_P_20_PCT_50_) g of purified condensed tannins per kg DM diet

**Table 7:** Effect of supplementing diets with purified condensed tannins on Bapedi ram meat attributes

Variable	Diet^#*^			
	GH_80_P_20_PCT_0_	GH_80_P_20_PCT_30_	GH_80_P_20_PCT_40_	GH_80_P_20_PCT_50_
pH				
Breast	5.9 ± 0.11	6.0 ± 0.01	6.0 ± 0.01	5.9 ± 0.21
Rump	5.7 ± 0.02	5.6 ± 0.11	5.7 ± 0.01	5.7 ± 0.01
Shank	5.4 ± 0.02	5.4 ± 0.01	5.4 ± 0.01	5.3 ± 0.11
Shoulder	5.6 ± 0.11	5.6 ± 0.10	5.8 ± 0.11	5.7 ± 0.09
Meat colour (rump)				
Lightness	49.3 ± 0.96	47.7 ± 0.85	48.0 ± 0.77	49.0 ± 0.82
Redness	10.3 ± 0.10	8.7 ± 0.10	15.3 ± 0.10	14.3 ± 0.10
Yellowness	5.7 ± 0.95	5.8 ± 0.81	5.7 ± 0.71	6.3 ± 0.90
Meat attributes				
Tenderness	3.7 ± 1.53	4.3 ± 1.58	4.0 ± 1.30	3.3 ± 1.15
Juiciness	3.3 ± 0.58	3.0 ± 1.00	3.3 ± 0.58	3.3 ± 0.58
Flavour	3.6 ± 0.33	3.7 ± 0.33	3.7 ± 0.33	3.7 ± 0.33
Overall acceptability	4.0 ± 0.02	4.0 ± 0.08	4.0 ± 0.03	4.0 ± 0.03
Shear force (N)	29.7 ± 4.04	32.5 ± 2.30	31.8 ± 1.42	29.9 ± 2.06

^*^Mean ± standard error (SE)

^#^Treatments were supplementation with 0 (GH_80_P_20_PCT_0_) 30 (GH_80_P_20_PCT_30_), 40 (GH_80_P_20_PCT_40_) or 50 (GH_80_P_20_PCT_50_) g of purified condensed tannins per kg DM diet

The effects of supplementing diets with purified condensed tannins on antioxidant activity in the meat of male Bapedi ram are presented in Table 8. Meat from male Bapedi rams on diets containing 30, 40 or 50 g of purified condensed tannins per kg DM had higher (*P* < 0.05) antioxidant activities than those from rams on a feed having no purified condensed tannins. However, meat samples of male Bapedi rams fed diets having 30, 40 or 50 g of purified condensed tannins per kg DM had similar (*P* > 0.05) antioxidant activities.

**Table 8:** Antioxidant activities in meat extracts of male Bapedi rams using DPPH assay

Treatment^#^	Antioxidant activity^*^
GH_80_P_20_PCT_0_	20.6^b^ ± 1.89
GH_80_P_20_PCT_30_	31.1^a^ ± 2.99
GH_80_P_20_PCT_40_	36.1^a^ ± 2.96
GH_80_P_20_PCT_50_	31.2^a^ ± 2.98

^a,b^Means in the same column not sharing a common superscript are different (*P* < 0.05)

^#^Treatments were supplemented with 0 (GH_80_P_20_PCT_0_) 30 (GH_80_P_20_PCT_30_), 40 (GH_80_P_20_PCT_40_) or 50 (GH_80_P_20_PCT_50_) g of purified condensed tannins per kg DM diet

^*^Mean ± standard error (SE)

## Discussion

Diets contained similar energy and protein levels of 18 MJ/kg DM and 13%, respectively. Thus, the diets met the recommended nutrient requirements for slow-growing sheep (NRC, 2007). Diets had similar nutrient contents except purified condensed tannins ranging from 0 to 50 g/kg DM. These diets were, therefore, appropriate for the determination of condensed tannin levels for optimal performance and reduced methane emission in yearling male Bapedi sheep. It was expected that the diets would have similar intake and digestibility values (McDonald et al., 2011) provided that condensed tannin supplementation did not have any adverse effects. Indeed, supplementing diets with purified condensed tannins did not affect the intake, digestibility and weight gain of male Bapedi sheep. It is possible that the purified condensed tannin amounts were not too high to adversely affect the intake, digestibility and weight gain of male Bapedi sheep. Al-Dobaib (2009) also reported that lambs fed diets having different levels of quebracho tannins had similar intakes and weight gains. However, other studies showed a decrease in intake and digestibility of nutrients when condensed tannins were incorporated into the ruminant diets (Makkar et al., 2007). The binding ability of tannins to microorganism cell walls adversely affects enzyme secretion and nutrient transportation within the organisms (McSweeney et al., 2001); thus, nutrient digestibility decreases because rumen microorganisms are not able to optimally ferment the diets. However, other studies found that condensed tannin inclusions in the diets had beneficial effects on the intake and weight gain of ruminant animals so as methane emission reduction (Min et al., 2003; Soltan et al., 2012; Ku-Vera et al., 2020; Cardoso-Gutierrez et al., 2021). According to Jayanegara et al. (2015), the inclusion of tannins from plant extracts, in ruminant diets, has been shown to decrease above 20 g/kg CH_4_. Moreover, Goel and Makkar (2012) reported that up to 50% of CH_4_ synthesis from ruminal fermentation has been in response to tannin or plant extracts with polyphenolic compounds. Molina-Botero et al. (2019) stated that antimicrobial properties present in tannin from plants have shown to decrease CH_4_ production, whereas Rira et al. (2015) reported that in an in vitro study 30 and 60% of *Acacia cyanophylla* supplementation resulted in a reduction of 37.5 and 56.2% methane production. However, the authors stated that this was due to the high content of condensed tannins concentrations present in *Acacia cyanophylla*.

Albores-Moreno et al. (2018) reported that *Leucaena leucocephala* rich in CTs supplementation in an in vitro study on diets for cattle decrease CH4 production up to 15.6 to 31.6% due to increased proportions of propionic and butyric acids. Moreover, Tan et al. (2011) also evaluated *Leucaena leucocephala* rich in CTs supplementation at 15 mg of CT/500 mg DM and observed a 47% reduction of methane production by cattle. Carulla et al. (2005) reported that *Acacia mearnsii* supplementation at 12.5 mg CT/500 mg DM resulted in 12% CH_4_ emissions reduction in sheep. The inclusion of tannins directly from plants or as plant extracts, in ruminant diets, has been shown to decrease CH_4_ above 20 g/kg.

Diet supplementation with purified condensed tannins reduced methane emission by Bapedi rams, by 51 to 60%. A 49.08 g supplementation of purified condensed tannins per kg DM diet was calculated, with the use of quadratic equations, to optimize methane emission reduction by male Bapedi sheep. This indicates that condensed tannin levels used in the study were enough to inhibit methanogenesis (Makkar et al., 2007). Carulla et al. (2005) observed a 12% reduction in methane emission when sheep were fed diets having different levels of condensed tannins. The authors indicated that tannins reduced methane production by decreasing the synthesis of acetate during digestion. Woodward et al. (2001) reported a 20 to 24% decrease in methane emission when sheep were fed diets containing condensed tannins. The present study indicates that purified condensed tannin supplementation levels of 30, 40 and 50 g per kg DM diet resulted in similar methane emission levels by sheep; possibly meaning that the levels used were within the required effective amounts for inhibition of methanogens in sheep. Mbanzamihigo et al. (2002) observed similar methane emission amounts by sheep when fed diets with tanniniferous legume supplementation levels of 0.16, 0.35, 0.41 or 0.51 g/kg DM.

Nutritional problems can be detected in the blood of animals (Ajao et al., 2013). In the present study, purified condensed tannin supplementation levels of 0, 30, 40 or 50 g per kg DM diet did not affect the blood parameters of male Bapedi sheep. Amounts of purified condensed tannins used induced no adverse effects in the animals. Haemoglobin values observed in this study were within the normal range of 8.15 − 10.75 g/dL for healthy sheep (Daramola et al., 2005; Akinyemi et al., 2010; Akinrinmade and Akinrinde, 2012). The present findings are similar to those of Dey et al. (2008) and Pathak et al. (2013). Purified condensed tannin supplementations did not affect haematocrit values of male Bapedi sheep, indicating that the animals were not anaemic (Purves et al., 2003). These results are similar to those of Solaiman et al. (2010) and Brown et al. (2016) for goats fed various levels of tanniniferous *Sericea lespedeza* and *Vachellia karroo* diets, respectively.

There were no significant variations in white blood cell (WBC) counts of Bapedi sheep on diets differing in purified condensed tannin supplementation levels, indicating that the animals were in good health (Ahamefule et al., 2008; Solaiman et al., 2010). Brown et al. (2016) observed no differences in WBC counts of indigenous Bapedi goats fed diets differing in tannins.

Purified condensed tannin supplementation did not affect the blood urea values of male Bapedi sheep. The blood urea values are within the range of a healthy sheep (Daramola et al., 2005; Akinyemi et al., 2010). Serum glucose values of male Bapedi sheep were not affected by dietary supplementation with purified condensed tannins, and they are within the normal range for a healthy sheep (Naumann et al., 2017; Pathak et al., 2013; Midaoui and de Champlain, 2005).

Results of the present study indicate that cholesterol levels were similar among Bapedi sheep fed diets with different levels of purified condensed tannins. These cholesterol levels, ranging from 2.42 to 3.3 mmol/L, were within the normal values for a healthy sheep (Naumann et al., 2017; Siri-Tarino et al., 2010). Olafadehan et al. (2014) observed no differences in cholesterol levels when goats were fed diets high in condensed tannins. Supplementation with purified condensed tannins did not affect the blood protein indices of male Bapedi sheep. The blood protein values were within the range of a healthy sheep (Naumann et al., 2017; Jain, 1986).

Purified condensed tannin supplementation levels used in the present study did not affect serum enzyme levels of Bapedi rams, suggesting that the animals were in good health (Thapa and Walia, 2007). Dey et al. (2008) observed no significant effects on aspartate transaminase and alanine transaminase in lambs fed diets with different levels of tanniniferous *Ficus infectoria* leaves. Purified condensed tannin supplementation levels did not affect blood electrolytes and mineral values of Bapedi rams. The electrolytes and mineral values obtained were normal for healthy sheep (Naumann et al. (2017), possibly meaning that the amounts used did not adversely affect mineral absorption in the gastrointestinal tract of the animals (Naumann et al. (2017; Sowande et al., 2008).

Purified condensed tannin supplementation did not adversely affect male Bapedi meat pH, sensory attributes and shear force values. The amounts of 0, 30, 40 or 50 g of purified condensed tannins per kg DM diet did not affect the end products of digestion and hence no differences in meat attributes of the sheep were expected. Dentinho et al. (2020) observed similar results when lambs were fed diets having different tannin levels. Similarly, Brown and Ngámbi (2018) observed no differences in goat meat attributes when diets differing in *Vachelia karroo* leaf meals were fed to goats. However, Luzardo et al. (2019) and Luciano et al. (2009) observed that tanniniferous feeds improved the meat colour of sheep. Gesteira et al. (2018) concluded that lamb meat tenderness decreased and shear force values increased as dietary tannin levels increased. Kobue-Lekalake et al. (2009) and Priolo et al. (2000) reported that meat from lambs on diets with lower tannin levels had better taste. The present study indicates that purified condensed tannin supplementation levels did not affect Bapedi sheep meat pH values which were within the range for a healthy sheep (Mostert, 2007). Priolo et al. (2002), also, observed that consumption of tanniniferous feeds by sheep did not affect meat pH values. However, other authors reported increased meat pH values when sheep were supplemented with purified condensed tannins (Ngambu et al., 2013).

Purified condensed tannin supplementation levels of 30, 40 or 50 g/kg DM diet increased antioxidant activity values in ram meat. Sufficient uptake of dietary antioxidants has been observed as an effective method to prevent lipid oxidation and avoid oxidation of cells and tissues caused by excess free radicals and oxidative stress (Negukhula, 2010). High antioxidant activity values in meat increase the quality and shelf-life of meat by reducing lipid oxidation (Fernandez-Gines et al., 2005; Karami et al., 2011; Juarez et al., 2012; Kasapidou et al., 2012; Karre et al., 2013). Marume et al. (2012) found that dietary supplementation with tanniniferous *Acacia karroo* leaf meal improved goat meat sensory attributes. Although poorly understood, the evidence suggests that condensed tannins can induce an antioxidant effect in living animals and their products through direct and indirect mechanisms, which can occur in an integrated and synergic way involving the following: (i) absorption of condensed tannins with low molecular weight or metabolites, despite condensed tannins poor bioavailability; (ii) antioxidant action on the gastrointestinal tract and (iii) interaction with other antioxidant agents (Soldado et al., 2021).

## Conclusion

It is concluded that condensed tannin supplementation levels of 0, 30, 40 or 50 g/kg DM diet had no effect on diet digestibility, growth and Bapedi ram meat sensory attributes. However, condensed tannin supplementation levels used in this study increased antioxidant activity values in male Bapedi ram meat. Long-term studies are suggested to ascertain the present results.

## Data Availability

Data and materials are available.
